# Early mobility to improVE health outcomes following cardiac surgery (EVE) clinical trial development: findings from workshop 1—embodied experiences of early mobility

**DOI:** 10.3389/fresc.2026.1801092

**Published:** 2026-10-08

**Authors:** Emily K. Phillips, Jacqueline L. Hay, April Gregora, Kathy Smith, Anna M. Chudyk, Carly Shaski, Sarah Gilchrist, Andrew Fagan, Maureen C. Ashe, Annette S. H. Schultz, Rakesh C. Arora, Todd A. Duhamel, Sheila O’Keefe-McCarthy

**Affiliations:** 1 Applied Health Sciences, Faculty of Graduate Studies, University of Manitoba, Winnipeg, MB, Canada; 2 Faculty of Kinesiology and Recreation Management, University of Manitoba, Winnipeg, MB, Canada; 3 Institute of Cardiovascular Sciences, St. Boniface Hospital Albrechtsen Research Centre, Winnipeg, MB, Canada; 4 Person with Lived Experience Partner, University of Manitoba, Winnipeg, MB, Canada; 5 College of Pharmacy, Rady Faculty of Health Sciences, University of Manitoba, Winnipeg, MB, Canada; 6 St Boniface Hospital, Winnipeg, MB, Canada; 7 Max Rady College of Medicine, Rady Faculty of Health Sciences, University of Manitoba, Winnipeg, MB, Canada; 8 Department of Family Practice, The University of British Columbia, Vancouver, BC, Canada; 9 College of Nursing, Rady Faculty of Health Sciences, University of Manitoba, Winnipeg, MB, Canada; 10 Department of Surgery, Northwestern University, Feinberg School of Medicine, Chicago, IL, United States; 11 School of Nursing, Faculty of Applied Health Sciences, Brock University, St. Catharines, ON, Canada

**Keywords:** arts-based research method, body mapping, cardiac surgery, early mobility in intensive care unit, intensive care, people with lived and living experience

## Abstract

**Introduction:**

Using arts-based research methods to engage people with lived experience (PWLE) supports the exploration of embodied knowledge, including senses, expressions, and emotions. Understanding embodied experiences may support closing the know-do gap by reducing the disconnect between research production and its translation into clinical care. Most research focuses on medical perspectives and less on patient experiences with early mobility after cardiac surgery (CS). No research explores patient experiences using arts-based research methods, which limits the depth of the exploration of their experiences. This article describes the findings of a qualitative, arts-based research method study.

**Methods:**

Body mapping was used to explore the embodied knowledge of early mobility in the CS intensive care unit (ICU). Body mapping involves participants creating a life-size tracing of a body in a position of their choice. The tracing is then filled with various art media, reflecting participants' lived experiences in response to the questions posed by the researchers. Three workshop sessions were held during which 1) the project, team, and body mapping process were introduced; 2) participants created body maps; and 3) body maps and the participants' experiences were discussed and co-analyzed by participants and researchers using compositional interpretation. Video recordings of the discussions were further analyzed using narrative inquiry and participant observation to build on the initial analysis.

**Results:**

Eight patients and two care partners participated. Five themes were generated that described the significant emotional and mental impact of mobility on patients following CS: Making Meaning of Movement, the Paradox of Movement, Moving Through the Emotions, Person-First, and Attitude.

**Discussion:**

These embodied experiences can inform novel interventions or approaches to enhance clinical care and research, thereby supporting successful mobilization and potentially improving patient health outcomes.

## 1 Introduction

In patient engagement in research (PER), people with lived experience (PWLE) of the health issue being studied are meaningfully and actively involved throughout the research process (1–3). Internationally, PER is recognized as important in generating knowledge to improve the healthcare system (1–3). Verbal communication methods have dominated PER thus far (4, 5). This dominance limits the knowledge available to the research process to that which can be put into words. Arts-based research methods use art throughout the research process. Examples of forms of art that can be used include photo-elicitation (6–8), poetry (8, 9), theatre (8, 10), dance (11), and visual art (12). Arts-based research methods broaden the knowledge gained from PWLE to include embodied knowledge, which cannot be conveyed through words alone (5, 12–15). Embodied knowledge can be defined as knowledge gained from and residing in the body (15). It includes elements of senses, expressions, and emotions (5, 12–15). Our narrative review explored how arts-based research methods have been used for PER and shared artistic methods, research stages, levels of engagement, and impacts, along with examples of how to use various artistic methods for PER (5). Using arts-based research methods for this purpose has been associated with a deeper understanding of the lived experience through their embodied knowledge (5). Arts-based research methods were further noted as potentially effective for flattening the power dynamic between PWLE and researchers and reducing the sense of tokenism that can occur in PER (5).

Understanding the experiences of PWLE and integrating these into interventions may align the conceptualization and understanding of phenomena among researchers, clinicians, and patients and support closing the know-do gap (1, 16). The know-do gap is the disconnect between research production and its translation into clinical care (16–18). For example, research findings are often applied to only 60% of patients 15 years after publication (18). A specific example for this study is the concept of early mobility following cardiac surgery (CS) while in the intensive care unit (ICU). Early mobility interventions have been associated with improvements in patients' physical health outcomes following CS (19–21). They have also been shown to be safe for patients after CS in the ICU (22–27) and are recommended in guidelines (28, 29). Despite its benefits, safety, and recommendations, integration of early mobility into CSICU clinical care is inconsistent (30, 31).

Importantly, embodied experiences of PWLE regarding early mobility in the CSICU are largely absent from the global literature. This limits the ability to understand and incorporate this valuable source of knowledge into interventions to align conceptualization among stakeholders and support their use in clinical care. Using arts-based research methods to explore embodied experiences of early mobility in the CSICU may help close the know-do gap by aligning the conceptualization of early mobility among researchers, clinicians, to consider this from the patient perspective (1, 16).

An arts-based qualitative study, using body mapping (32–36) to engage PWLE who have undergone CS and been admitted to a CSICU, was undertaken. Bodies are an essential source of knowledge (15). Body mapping was first used in research in the early 2000s, investigating the experiences of people from South Africa living with HIV (32, 33, 35, 37). Since then, body mapping has been used to examine sources of strength and support for people in treatment for problematic alcohol and other drug use (33), drugged bodies and injecting drugs (37), with youth exploring how sexual and reproductive health relates to their social situation (35), the experiences of women with mental health disorders or psychosocial distress (38), with marginalized women (34), and body image and premenstrual changes to eating and exercise practice (36). To our knowledge, this is one of the first studies to use body mapping to explore experiences of early mobility in the CSICU.

Body mapping involves each person creating a life-size tracing of a body in the position of their choosing (32–36). Each tracing is then filled in based on personal reflections on questions posed in the workshop (32–36). Their body map can be filled in with paint, markers, crayons, collages, or words in whatever way is meaningful to the PWLE, creating the body map. The final step is for each PWLE to describe their body map and the meaning behind their chosen representation of their experience (32–36). This diversity in how reflections are shared supports the shift in power from researchers to PWLE, as PWLE determine how they will share their knowledge (32–36). The combination of creativity and reflection (33–36, 38) leads to a deeper, embodied self-awareness and understanding of identity within a lived experience (34).

Body mapping may reduce the power imbalance between researchers and PWLEs and the sense of tokenism that can exist in PER (34), thereby fostering collaboration and meaningful engagement (5). PWLE control what and how they share their experience, as there is no “right way” to create a body map (35, 36). For example, PWLE can position their bodies how they wish, use or exclude anatomic features, and use photographs, words, colours, and their placement to tell their story (36, 38). PWLE can collaborate and make body maps together or create them individually (39). Finally, body maps can include multiple, smaller bodies depicting several aspects of an experience or story (37). This expressive freedom supports PWLE in owning both their experience and how it is shared (5). By co-analyzing the body maps, as this study did, PWLE also co-own the interpretation of their experience and how it is shared (5). A similar process has been used in other body mapping studies and has been described and more deeply explored conflicting feelings around gender (35). In this way, sharing body maps through guided reflection and discussion among all team members creates a space for co-creating transformative embodied knowledge, which is deeper and more holistic than a discussion alone (12, 13). By inviting PWLE to share their experiences through body mapping, the experience and understanding of early mobility in the CSICU flows from embodied knowledge and meaning of mobility as they lived/experienced it.

The objective of this research was to co-create an understanding of the embodied experience of early mobility in the CSICU. This is the second article in a three-part series that describes a two-phase study to develop a PWLE-informed understanding of early mobility in the CSICU. The methods are reported in an article that shares our novel workshop model (40). Phase 2, reported in a separate article, employed two didactic consultation workshops and the use of nominal group technique to co-create a definition of early mobility, identify barriers and facilitators to early mobility, and select outcome and experience measures for use in future intervention research (41). Collectively, this work is part of the Early mobility to improVE health outcomes following cardiac surgery (EVE) Clinical Feasibility Trial Development study.

## 2 Methods

### 2.1 Research design

This work engages PWLE as co-producers of research using an arts-based research method (body mapping). Reporting of this work follows the updated Guidelines for Reporting Involvement of Patients and the Public (GRIPP-2) long form (42) and the Standards for Reporting Qualitative Research (43). See the supplemental material for the checklists.

### 2.2 Research paradigm

Engaging PWLE in research requires researchers to believe that reality can differ across experiences and contexts, and to ask questions and seek results relevant to the population of interest (5). Such beliefs align with a pragmatic worldview, through which this study was conducted (44–46). Through such a worldview, knowledge is contextual and is derived from experience (46, 47). This requires engaging PWLE throughout the research process and privileging their embodied knowledge. Research through a pragmatic worldview also focuses on producing research findings relevant to research users (44–46). This aligns with the tenets of PER (16). Within this worldview, the best approach to gaining knowledge is preferred over reliance on a specific research methodology (44–46). This supports the use of arts-based research methods for PER, as they can elicit embodied knowledge (5).

### 2.3 Context, researcher characteristics, and reflexivity

This study occurred at St Boniface Hospital, a tertiary hospital in Winnipeg, Manitoba, Canada (detailed elsewhere) (31). All CS (∼1,000 annually) in Manitoba are performed at this site, with the majority of procedures including coronary artery bypass grafting (CABG), valve surgery, or a combination of the two (31).

Our diverse research team included a former CS patient (AG), a care partner of a former CS patient (KS), a registered nurse who formally worked in the CSICU and doctoral candidate (EKP), a current CSICU registered nurse (CS), a program director with clinical expertise as a critical care nurse (SG), two cardiac surgeon/intensivists (AF & RCA), a patient-oriented researcher (AMC), a physiotherapist with expertise in early mobility (MCA), a clinical exercise physiologist and doctoral candidate with exercise intervention development experience (JLH), a nurse scientist with research expertise in engaged research approaches (ASHS), a nurse scientist with expertise in critical care and arts-based research methods (SOM), and a PhD kinesiology professor (TAD).

Several research team members' CSICU experiences and roles are provided to clarify the intersection of personal, clinical, and research interests that led to this study, which are important when considering the power differential from an ethical perspective. EKP has not worked clinically with patients in the CSICU or across the broader CS journey since 2018. RCA has not worked clinically in the CSICU at the study site since 2021. AF and CS currently work clinically in the CSICU.

### 2.4 PWLE recruitment and equity, diversity, and inclusion considerations

PWLE were recruited from a database of previous CS patients who had consented to be contacted for future research. Inclusion criteria were adults over 18 who have undergone or had a loved one undergo outpatient coronary artery bypass grafting, valve, or a coronary artery bypass grafting and valve procedure in the last five years, cognitively able to participate in consultation workshop, had access to the internet and an electronic device, were willing to reflect on personal experiences relating to CS and share these in a group setting, and consent to be recorded. We aimed to engage 10 PWLE to align with recommendations for group size to facilitate discussion and participation (48). The inclusion of care partners was suggested by the PWLE co-authors, who shared the difficulty of remembering the ICU hospitalization and the key role care partners play in helping to piece together memories of the time.

The small group size and the database of patients willing to be contacted for future research limited the potential diversity of our study. Due to these constraints, diversity was prioritized in 1) self-identified gender, 2) patients and care partners, and 3) urban and rural representation. We prompted inclusion through confidential, personalized discussions with each participant prior to the first workshop session, where any required supports were identified. For example, we met one participant in the parking lot with a wheelchair and assisted them to the workshop location due to mobility restrictions. In this way, we supported equitable access to the workshop sessions to promote the ability to participate as required. PWLE demographics were collected at the beginning of Workshop 1 Session 1 using a questionnaire. Demographics collected included age, their self-identified ethnicity, whether they are a patient or care partner, length of stay in CS ICU post-cardiac surgery, type of cardiac surgery (valve, CABG, valve and CABG, or other), and the year they underwent cardiac surgery.

### 2.5 Patient engagement approach, levels and stages of engagement, and compensation

The approach to engaging PWLE was informed by the Canadian Institutes of Health Research Strategy for Patient-Oriented Research Framework (1) and team members' PWLE-engaged scoping review methods and consultation workshops (49, 50). A multi-level engagement approach was used. The levels of engagement are defined by the International Association for Public Participation's Spectrum of Public Participation (51). This spectrum defines engagement along a continuum of increasing impact on research decision-making, from “inform” to “empower” (51). Two PWLE (AG and KS) were engaged at the level of collaborate (51), which is in the middle of the spectrum and is described as “to partner with the public in each aspect of the decision, including the development of alternatives and the identification of the preferred solution” (51). These PWLE partners contributed expertise as members of the research team at all stages of the research through meetings, developing the body mapping workshop plan, co-facilitating the workshops, and co-analyzing the workshop findings (4). Additionally, the PWLE that participated in the body mapping workshops were also engaged at the level of consult, which is described as gaining public feedback (51), during data collection and analysis (4). Engaging PWLE at multiple levels enabled this work to be fulsomely guided by lived experiences. Collaborating with PWLE as members of the research team ensured the design and conduct of the study were informed by lived experiences. Consulting additional PWLE included a broader range of perspectives to inform the study objectives than including only the PWLE partners. It also supported data collection and analysis led by PWLE.

The levels of engagement informed the compensation provided (52). The PWLE collaborators (AG and KS) were compensated $25 per hour for their time co-facilitating and co-analyzing. The PWLE consultants were also compensated $25 per hour for their time. All out-of-pocket expenses were reimbursed, including mileage and transportation.

### 2.6 Ethical considerations

This work was conducted in alignment with the Tri-Council Policy Statement: Ethical Conduct for Research Involving Humans—TCPS 2 (53). Institutional review board approvals for the case study exemplar were obtained in March 2024 from the University of Manitoba Research Ethics Board 1 (HE2024-0035) and the St Boniface Hospital Research Review Committee (RRC/2024/2158). All PWLE reviewed and signed a consent form prior to participating in the workshops. PWLE were made aware that consent was an ongoing process, and they could withdraw consent until data analysis began, at which point it was no longer possible to remove their data from the study. No PWLE withdrew their consent.

### 2.7 Body mapping

Body maps were created and partially co-analyzed over three three-hour-long in-person workshop sessions in June 2024 (Figure 1). These workshops have been described in detail elsewhere (40). Briefly, in the first session, PWLE were introduced to one another, the workshop facilitators (SOM, EKP, JLH, AG, and KS), the project, and body mapping. This occurred through facilitated in-person discussions and the sharing of mobility experiences from the PWLE perspective post-CS. All PWLE attended the first session. For the second and third sessions, PWLE were split into two groups of five to allow the facilitators to support them appropriately. In the second session, body maps were created. The third session involved PWLE sharing their body maps and experiences in a facilitated video and audio-recorded discussion with other PWLE and the workshop facilitators. This is when the co-analysis of the body maps began. This collaborative process aimed to begin creating a story from the experiences shared by PWLE (12). Each PWLE was asked to talk about their journey with early mobility in the CSICU and about what they included on their body map. They were asked to reflect on their choices for how they represented their experiences through images, textures, colours, and words. Finally, they were asked what the title of their body map was and why they chose it. These recordings were transcribed to allow for a deeper analysis of discussions.

**Figure 1:**
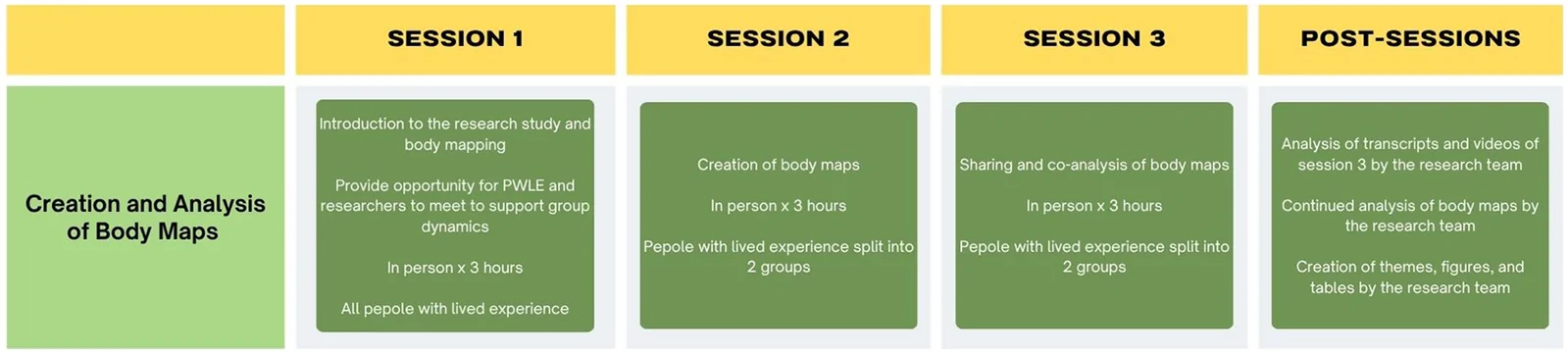
Visual overview of the components of the three workshop sessions.

### 2.8 Data analysis

PWLE demographics were described conventionally. Three data sources were analyzed: the body maps, the transcripts of the body map presentations, and the videos of the presentations. Analysis began with compositional interpretation, a visual analysis of the body maps (54). It then expanded to a narrative inquiry analysis of the stories shared in the transcripts (12). This step built upon initial themes and categories from the body maps and added new themes. Finally, participant observation, noting body language, reactions, and expressions in the video recordings (55) was used to confirm and expand emerging themes and categories. The data analysis approach enabled data and investigator triangulation, as multiple research team members independently analyzed data (EKP, SOM, AG, KS, & JLH). Analysis also involved exploring the production of the image by including PWLE in data analysis and interpretation.

#### 2.8.1 Compositional interpretation

Body map analysis began during session three using compositional interpretation, which focuses on the image created and its impact through content, colour, spatial organization, light, and expressive content (54). The analysis of colour includes the dominant hue, colour saturation, the value (lightness or darkness) of colours, whether the colours are harmonious, and what is stressed with colour (54. The analysis of the image's content includes describing what is shown and which symbols or icons are used (54). The analysis of spatial organization looks within the image at the direction of lines and if the image is static or dynamic and expands beyond the image to include the point of view the image is created from (54). The light is the use of light to highlight elements of the image (54). Finally, the expressive content of the image is the emotive effect of the image, what emotions the image makes one feel (54). These elements were included in the questions that guided the conversations where PWLE shared their body maps. The PWLE were asked why certain colours were chosen, what symbols represented, why they positioned their body in the manner they chose for their body map and so on. In this way, PWLE began the compositional interpretation, providing interpretations of their body maps and the meaning behind the included elements.

Following the third session, EKP, SOM, AG, KS, and JLH discussed the body map presentations, reviewed the body map photographs, and began coding. One PWLE co-author (AG) created a table of the interpretations by the PWLE. She outlined the elements of each body map and the meanings assigned to them by the PWLE. The other facilitators expanded this table, adding in interpretations that were missed or adding deeper descriptions. The first author (EKP) organized the expanded table into the elements of compositional interpretation. Light was not included in the compositional interpretation as no body maps included this element. The analysis then expanded to include the discussions that occurred when presenting the body maps.

#### 2.8.2 Narrative inquiry

Narrative inquiry focuses analysis on a larger scope of data, including plotlines, thematic structures, and social and cultural references rather than on the smaller scope of words and phrases more common in other qualitative methodologies (56). Narrative inquiry of workshop discussions allows for exploration and rich descriptions of shared experiences (12). The process for narrative inquiry follows the process of thematic analysis (12). First, two research team members (SOM and EKP) each read and reread the transcripts of the recordings of the PWLE discussing their body maps to gain familiarity. They then independently coded the same four transcripts and discussed the emerging narratives. The remaining four transcripts were then coded. There was a final discussion of the emerging narratives. The narratives were organized in a table that included names, descriptions, and examples. At this point, an overall structure was unclear, as the analysis was not yet complete.

#### 2.8.3 Participant observation

Nonverbal communication is important to consider, especially when PWLE are communicating emotions and experiences (57). Elements missing from transcripts, such as tone of voice and facial expression, account for over 90% of communication of emotions (57), so exploring these using participant observation of the videos supports a rich analysis. To ensure that PWLE's gestures and body language were captured and analyzed, the videos of PWLE presenting their body maps and of the other PWLE watching the presentations were also analyzed as a source of data by EKP and SOM. This analysis aimed to tie together the art created and the words used to describe the art.

The analysis of videos was guided by Spradley's nine dimensions of descriptive observation (55) (Table 1), and Figure 2 provides an overview of the process. Initially, a table was created that included each dimension. Two research team members, SOM and EKP, then watched the first video of a PWLE presenting their body map and analyzed each dimension independently. For instance, the room shown in the video was noted under Space, while PWLE's gestures during the discussion were recorded under Activities. As Spradley indicated, not all nine dimensions are relevant for every observation (55). In our observations, because the aspects of Space, Objects, Acts, Events, Time, and Goals were consistent, they were noted only during the first video review. As such, our focus was on observing Activities and Feelings. After reviewing the initial video, SOM and EKP compared and discussed their observations. This process was repeated with the second video, which was also watched concurrently. The remaining six videos were independently watched and analyzed by EKP and SOM, and once complete, they scheduled time to discuss their findings and emerging themes. The same approach was followed in analyzing the videos of PWLE in the audience as they watched others present their body maps.

**Table 1:** The nine dimensions of participant observation adapted based on Spradley (55).

Number	Label	Description
1	Space	The physical place where the observation occurred
2	Actors	The people/participants involved
3	Activities	A set of related acts that participants perform
4	Objects	Physical things present in the observation
5	Acts	Single actions participants perform
6	Events	Related activities participants perform
7	Timing	The sequence of acts or activities
8	Goal	What are participants trying to accomplish
9	Feeling	The emotions expressed

**Figure 2:**
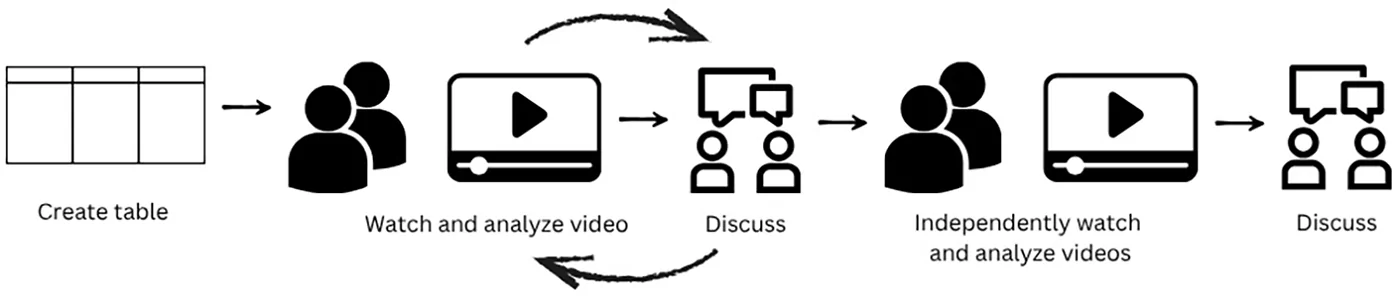
Visual overview of the process of participant observation.

#### 2.8.4 Data integration

The first author (EKP) conceptualized the structure of the overall experiences of early mobility by combining the findings of the three analyses and integrating the visual and verbal data. The analysis of participant observation was reviewed with the themes generated through narrative inquiry. Analysis of participant observations supported the understanding of the embodied experiences described and the associated emotions. Emphatic nodding, gestures, and facial expressions were elements of participant observation that clarified the themes generated through narrative inquiry. For example, although only one PWLE explicitly mentioned the need for a positive attitude, the responses of other PWLE noted during participant observation validated this as an important category. The compositional interpretation was reviewed, and elements were integrated into the descriptions of the generated categories. For example, the verbal descriptions of the need for a positive attitude were supplemented with visual elements from body maps. In this way, visual and verbal data were integrated in the descriptions of categories.

## 3 Results

A total of 10 PWLE, consisting of 8 patients and 2 care partners, participated in this study. We recruited 4 females/women and 6 males/men. The PWLE included one male/man care partner, one female/women care partner, 3 female/women patients, and 5 male/men patients. Demographic information can be seen in Table 2. The PWLE included 2 people self-identifying as Métis, of which 1 also self-identified as Ojibwe. 2 PWLE declined to share their self-identified ethnicity. The ages of the PWLE ranged from 59 to 82, with a median of 69 [interquartile range (IQR) 61-82]. Their median CSICU length of stay was 5 days (IQR 3-30).

**Table 2:** Demographics of PWLE participants.

Participant ID	Age	Sex assigned at birth	Self-identified Gender	Self-identified ethnicity	Role (Patient or care partner)	ICU LOS	Type of CS	Year of CS
P1	82	Male	Man	Irish	Patient	5 days	CABG + Valve	2021
P2	68	Male	Man	n/a	Patient	Unknown???	Valve	2022
P4	60	Female	Woman	White	Patient	6 days	CABG + Valve	2023
P5	63	Male	Man	Metis	Care Partner	6 days	CABG + Valve	2023
P6	70	Male	Man	Participant left blank	Patient	30 days	CABG	2023
P7	59	Female	Woman	Caucasian	Patient	3 days	Valve	2022
P8	80	Female	Woman	Canadian-French	Patient	7 days	valve	2023
P9	75	Male	Man	white/Caucasian	Patient	3 days	CABG	2023
P10	73	Male	Man	CDN	Patient	5 days	CABG	2023
P11	60	Female	Woman	Metis/Ojibwe	Care Partner	3 days	CABG	2023

### 3.1 Compositional interpretation

The compositional interpretation of the body maps is shared as: colour, content, spatial organization, and expressive content. The complete compositional interpretation can be found in Table 3. An overview is described below. Two examples of body maps can be seen in Figures 3,4.

**Table 3:** Compositional interpretation of the body maps of the lived experience of early mobility after cardiac surgery.

	Content and Colour							Spatial Organization	Expressive Content
All	Lines/ Tubes	Brain Fog	Attitude	Pain	Dizziness/ stability	Happy to be alive	Hallucinations	From perspective of another but describing their experience	
P1	Pipe cleaner	Written	Written	Unclear				Laying face up on back	
P2		Cotton balls/ zzzzzz		Back: dark red lines, blue coils Gout: red areas	Holding onto IV pole			Laying face up on back	Pain Confusion
P4/5	Pipe cleaner	Cotton balls				Background of bright colours, sun, happy face		Sitting facing left	Joy
P6	Pipe cleaner	Cotton balls/????	Negative/ depressed: black around head	Red scissors in back	Black rocks and light blue waves under feet			Sitting facing left	Pain Confusion Sadness
P7	Drawn in blue					Background (red heart, coloured flowers, sun, words)	Glued on bunnies	Laying face up on back, body cut out from rectangular paper	Pain Confusion
P8	Pipe cleaner			Back: squiggly red lines leading to back with pain written		Written in thought bubbles, heart stickers		Laying face up on back	Joy Pain
P9/11		?? Around head	Positive: written in bold words					Laying face up on back Arms in 2 positions Heart very large	Joy Confusion
P10	Drawn	X's & O's in ink		Legs: red squiggly lines				Sitting facing left	Confusion Pain

**Figure 3:**
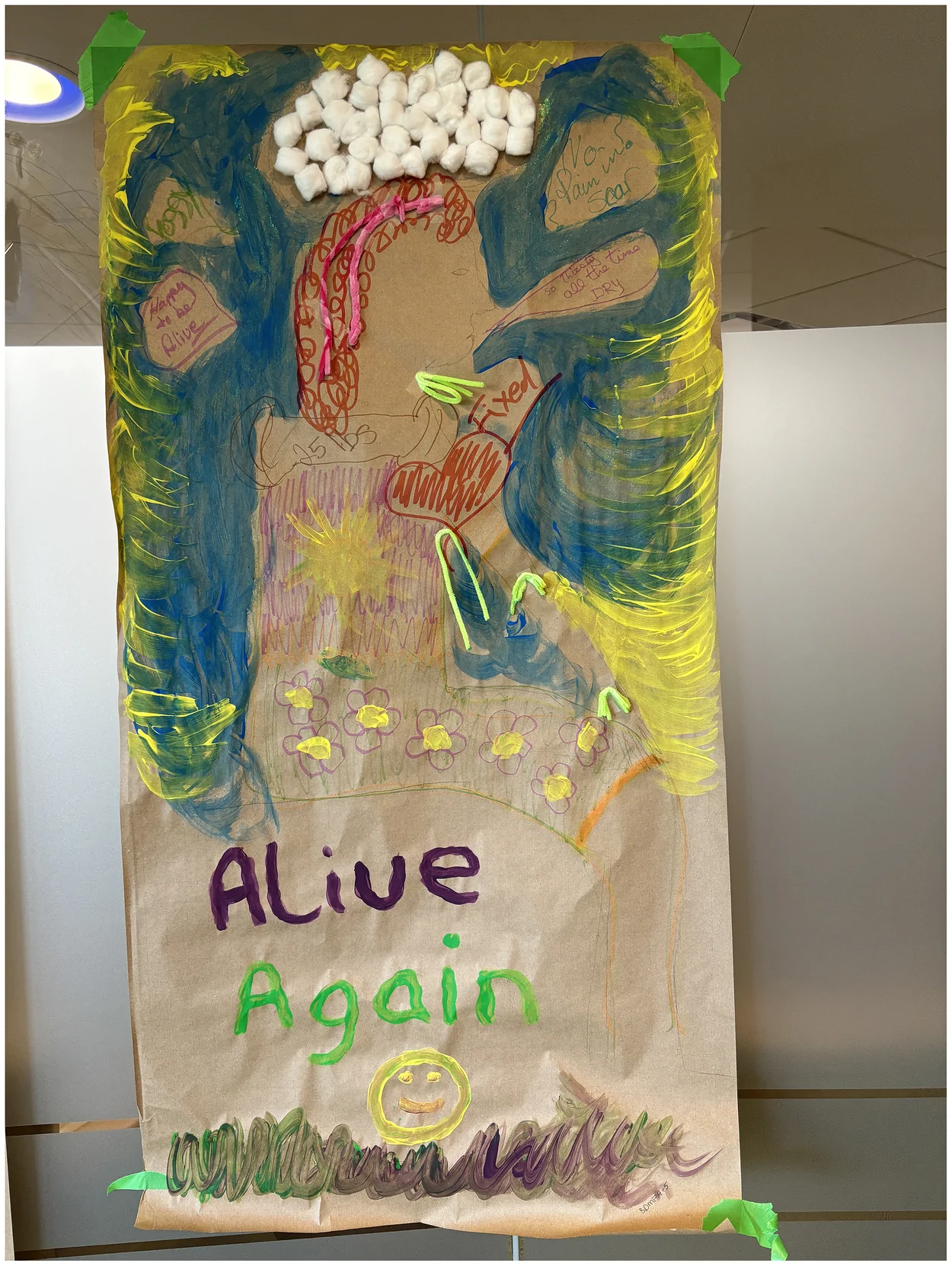
Body map created by P4 & P5.

**Figure 4:**
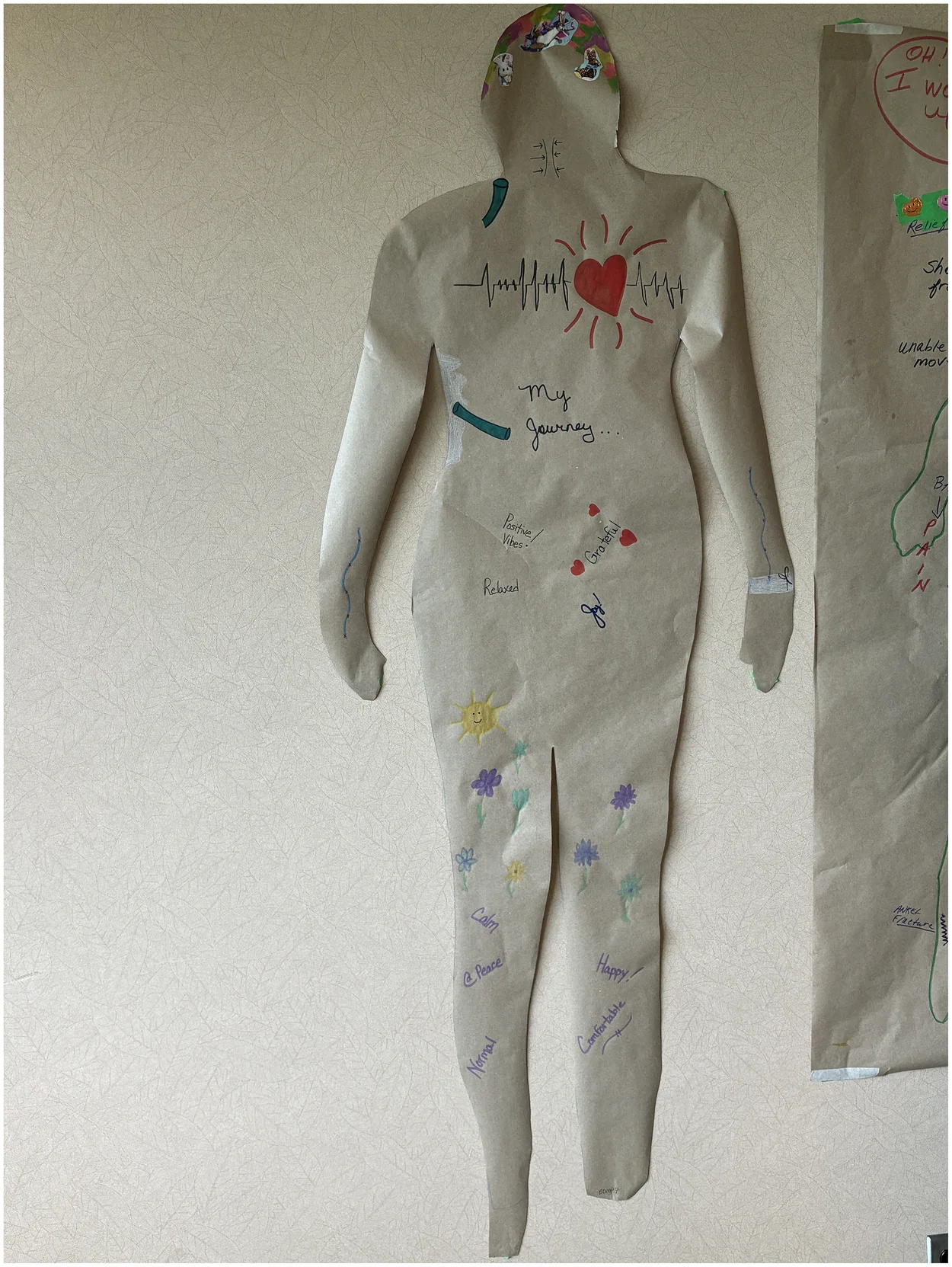
Body map created by P7.

#### 3.1.1 Colour

Vivid colours dominated all the body maps, such as rich reds and saturated black. Several of the body maps included vivid yellows, pinks, blues, and greens. The colours were harmonious both within each body map and among all the body maps. Participants described using colours to portray pain, happiness, joy, and depression and pieces of equipment, such as chest tubes and intravenous lines.

#### 3.1.2 Content

The content of the body maps included lines and tubes, brain fog, attitude, pain, dizziness or instability, being happy to be alive, and hallucinations. These were expressed mainly through symbols and icons. For example, pipe cleaners were used to depict lines and tubes. Cotton balls and question marks around the head were used to depict brain fog. Pain was depicted in several ways, such as scissors stabbing the lower back (P6) and red lines on the legs (P10). Rocks on the ground depicted a sense of instability (P6).

#### 3.1.3 Spatial organization

All body maps were organized anatomically with lines following a typical body shape. Three were sitting, all facing left, while five were standing or lying flat; it is not possible to differentiate between the two positions. One body map (P9) has arms in two positions, crossed across the chest and at the sides. This body map also included a large heart. This was described by P9 as both the pillow they used to splint their chest and as symbolic of his large heart that took everything from CS and recovered. All PWLE stated that their body map was their experience, but their experience is from another's perspective, not from their own.

#### 3.1.4 Expressive content

Four key emotions were noted in the body maps: joy, pain, sadness, and confusion. On the body map of P8, joy was expressed using bright, vivid colours and the inclusion of heart stickers around the words “Happy to be Alive.” Pain was expressed with vivid and saturated reds and blacks, the use of squiggly lines, and the word pain. Sadness was expressed in one body map, P6, by black around the head. Finally, confusion was expressed using cotton balls or question marks around the head.

### 3.2 Embodied experiences of early mobility

Five themes and twelve categories were generated from the three data sources. The themes are Making Meaning of Movement, the Paradox of Movement, Moving Through the Emotions, Person-First, and Attitude. These themes and the corresponding categories are described below. Quotations, examples from the body maps, and descriptions of the observations are included as exemplars to illustrate the themes. The quotations are taken from the PWLE's descriptions and explanations of their personal body maps. They are unedited. See Figure 5 for a visual overview of the themes and categories.

**Figure 5:**
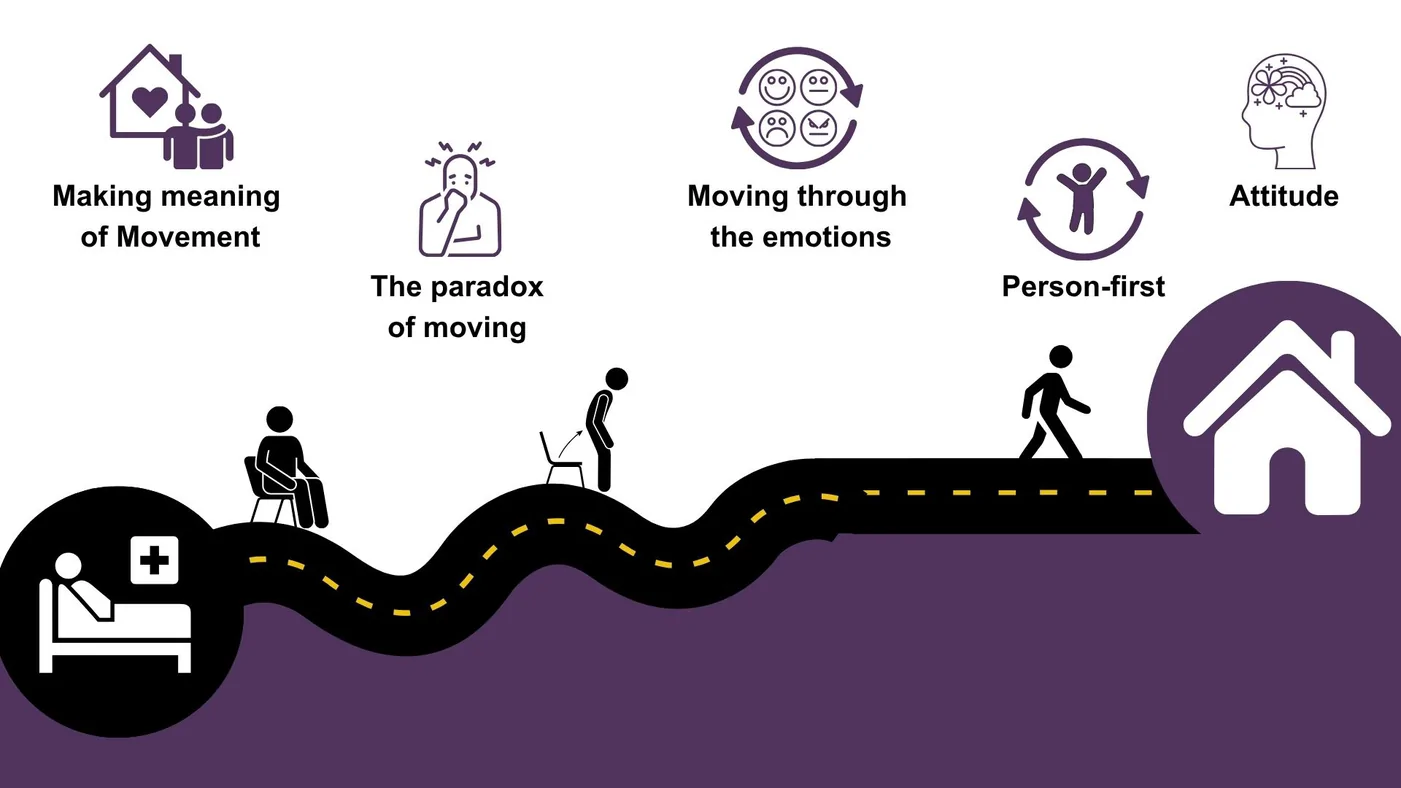
Overview of themes and categories.

#### 3.2.1 Making meaning of mobility

The theme of Meaning Making of Mobility was generated by PWLEs, who described mobility as more than just moving post-operatively. Three categories comprise this theme: movement means I survived, move to get home, and motivation to move. Mobility was regarded as a sign of survival. PWLE extended our current understanding of mobility and discussed it in relation to what their lives at home required. PWLE described what motivated them to move. These motivations related to what was important to them.

##### 3.2.1.1 Movement means I survived

When asked about moving after CS, PWLE often discussed moving as a sign of survival. This spanned the CSICU journey, from first waking after CS to survival in a broader sense, that they would be well again and able to move through their lives. Movement while waking following CS was perceived as an immediate sign that PWLE had survived their CS. PWLE frequently described waking from surgery and, before opening their eyes, moving their hands and feet to ensure they were alive. “Once I kinda woke up so to say, I moved my hands, arms, legs and I feel OK, I can…Everything is working yeah” (P1). Moving in the CSICU was a sign to PWLE that they had survived their cardiac journey. They could feel that the limitations on their bodies due to their cardiac issues were resolving. This signalled to them that they survived and could reclaim their lives and their identities through movement. This is seen in the title of P4/5's body map, “Alive Again.” See Figure 2.

##### 3.2.1.2 Move to get home

PWLE extended our current understanding of mobility and discussed it in relation to what their lives at home required of them. Multiple meanings were attached to the ability to move post-CS. For some, it was linked to being able to live the life they chose and that *movement* within that context was an integral part of living that life. One participant commented, “I…I knew that was going to help me get out of there faster and try to get back to my regular life” (P2). Another PWLE tied moving to the pragmatics of getting home: “Well…to get washed yourself and do all that that was a big treat. And then starting with the walker because people walked with me with the walker until I navigated it. And I felt OK, this isn't so bad and I'm in the house but it's only a bungalow and I thought I should be able to manage” (P8).

##### 3.2.1.3 Motivation to move

PWLE described what motivated them to move. These motivations related to what was important to them. Motivation was deeper than practicalities and being able to complete activities of daily living. It aligned with their personalities, their quirks, or their perceptions of a reward. These motivations were powerful tools to encourage and support mobility in the CS ICU. For example, one PWLE described how being able to get a sweet treat motivated him to mobilize:

“ …when I was in the ICU with the walker and…I did…the nurses were help me walk down the hall 30 or 40 feet yeah but every day I’d go farther and then when I found out there was ice cream that you can take, then I went all the way around the ice cream.” (P10).

#### 3.2.2 The paradox of movement

The paradox of movement simultaneously pushed PWLE to mobilize and yet pulled them toward sedentary behaviour. This theme is comprised of the categories of tensions experienced around moving and dealing with post-operative symptoms. The tensions around moving arise from knowing that one needs to move post-CS, but moving is problematic because ICU equipment and healthcare provider support are required. Symptoms were described through art on the body maps and through conversations, including difficulty sleeping, depression, difficulty swallowing, hallucinations, wobbly on feet, pain/ discomfort, fuzziness/ grogginess/ confusion. These symptoms led PWLE to avoid early mobility. See Table 3 for an overview of how symptoms were depicted on body maps.

##### 3.2.2.1 Tensions around moving

The Tensions Around Moving arise from knowing that one needs to move post-CS, but moving is problematic because of the ICU equipment and healthcare provider support required by previously fairly independent people. PWLE depicted ICU equipment on their body maps using pipe cleaners (P1, P4/5, P6, and P8). One PWLE, P2, included his intravenous pole with his body map, depicting how the ICU equipment became part of him during mobility. Other PWLE used words to describe the support they needed to move. “Two nurses come over to get you out of bed because of all these tubes and paraphernalia. They had to get this out and I'm only moving from here to where you're sitting at the sit in the chair…” (P1). Thus, PWLE perceived that the effort required to support them in getting out of bed was potentially higher than the benefits. This caused tension for them as they recognized the value of mobility in their recovery: “I'm glad I got kicked out of bed…I find that the more I get forced to get up the better I feel.” (P2).

##### 3.2.2.2 Symptoms

Symptoms were described through art on the body maps and through conversation. These symptoms were discussed as a barrier to where PWLE engaging in early mobility. For example, P6's placement of the rocks under their feet depicts how they experienced feeling unsteady on their feet while mobilizing. “Maybe little depressions on the ground for instability. When I first stood up after this, I was a little bit unstable I had to use the walker. I used a walker to get to the chair” (P6).

Several participants drew cotton balls around the heads on their body maps to depict brain fog (P2, P4/5, and P6) while others depicted brain fog with other symbols (P1, P6, P9/11, and P12). “You know, everything is fuzzy. Everyone said it is. Everybody, when you first wake up even…even all through that that first evening, that first night and for me anyway, until the next day, it was still…you were still foggy…” (P4).

Pain was depicted in red by P2, P6, P8, and P10. P4/5 depicted weights on their shouldersbut no pain. “Then when I did try to move, that's what this is about was the back pain. I could not get over the back pain, it took over all the other pain really” (P8).

#### 3.2.3 Moving through the emotions

Mobility after CS was explained as an emotional experience. Three categories fall under this theme: being restricted, happy to be alive, and redefining self. PWLE described oscillating from being frustrated and embarrassed about being restricted by medical equipment to being happy to be alive and taking this opportunity to redefine themselves through mobility. Emotions were written on the body maps and depicted using colours and through expressive content. PWLE figuratively moved through these emotions while moving after CS. Furthermore, these emotions were driven by or stemmed from mobility.

##### 3.2.3.1 Being restricted

Not being able to move freely, even in bed, due to feeling restricted, was frustrating for PWLE. This sense of being restricted seemed to move beyond the physical. The embarrassment and frustration that PWLE felt was seen and heard through participant observation. For example, one PWLE described being restricted as:

“You know, you can't move very much, you can't roll over this way to relieve your back, roll that way, you need help sitting up exactly help moving, going to chair or going to the bathroom. You need help. A little bit of frustration and embarrassment.” (P6).

This PWLE was gesturing side to side more aggressively than in other parts of the conversation when talking about rolling. Their tone of voice changed to be harsher and halting. When this PWLE said embarrassed, their eyebrows came together, and their voice softened. In this way, they both described and embodied the emotions of frustration and embarrassment which were tied to mobility.

##### 3.2.3.2 Happy to be alive

Negative feelings existed along with feelings of happiness at being alive after CS. “It's…it's my…my freedom of…of having…living again” (P4). Feeling happy to be alive was tied to mobility as this PWLE described this emotion when discussing how they could now move without feeling short of breath. This PWLE tied being able to get up and walk to being alive. Tying mobility to being happy to be alive is also seen in the expressive content of the body maps. For example, joy and happiness are expressed on the body maps through the background of bright colours, sun, and a happy face in P4/5 and the red heart, coloured flowers, sun, and words “calm”, “normal”, “at peace”, “happy!”, and “joy!” on the body map of P7. See Figures 2,3.

##### 3.2.3.3 Redefining self

Moving after CS made many PWLE pause and redefine themselves. They reflected on how the differences in moving after CS impacted them beyond mobility and how this carried through into their everyday lives both mentally as well as physically. As one PWLE noted, this was not due directly to physical limitations but rather reflection on the physical limitations.

Yeah it slowed me down. I don't…well, I still do things in a hurry, but not as fast as I used to…it did make me stop you realize that, you know, you don't have to rush everything. But that it really didn't have so much to do with my heart, like it wasn't a physical condition it was more of a mental…a mental condition (P9/11).

This PWLE included the phrases “less power walking” and “change of pace” on their body map to describe how moving after CS helped redefine who they are.

#### 3.2.4 Attitude

PWLE noted the importance of their attitude and how this impacted how they understood the value of early mobility after surgery and their willingness to be open to knowledge, support, and assistance from others. Two categories fall under this theme: positive attitude and if you know, you can endure. PWLE shared that their attitudes were driving factors in moving after CS, either driving them to move or to be sedentary. Being prepared for CS by knowing what to expect post-CS was important to PWLE. This helped them mentally plan for their recovery which helped them endure the recovery process and the symptoms they experienced.

##### 3.2.4.1 Positive attitude

Having a positive attitude towards early mobility was key to PWLE. “…went in with the, you know, my attitude is you have to have a good attitude. I believe the staff, you know doctors and the nurses and whatnot, they have attitude to come and care for you, you know. They don't know you, you don't know them, but if you win with a positive attitude, everything's gonna work out fine. That usually does, so that's important” (P1). This is also seen in the title of P1's body map, “Attitude is Everything.” The value of a positive attitude was noted in participant observation. As P1 was sharing their body map and discussing the above quote, P2 was nodding emphatically in agreement.

##### 3.2.4.2 If you know, you can endure

Knowing what to expect related to early mobility helped PWLE prepare for it. “I was informed of that (pain from surgery when moving). I was aware of that there was a period of time, which wasn't that long, that I knew I'd have to endure and I could. I felt I could overcome it. I was aware of it ahead of time and as expected, so big deal. That was of no surprise and then I was prepared for it” (P2). Knowing what to expect went beyond symptoms and included knowing about the importance of mobility. “I knew I had to start moving. If you don't move, you lose it” (P8). It also related to how mobility was key to being discharged from the hospital: “My attitude was, I want you to check off all the boxes so I can go home…if I have to be mobile, I’ll be mobile, if I have to do this, I'll do that…” (P2).

#### 3.2.5 Person-first

Being seen, understood, and treated like an individual rather than another surgical patient helped to get PWLE moving. This theme includes the categories of individualized care and support and kindness from care partners and healthcare providers. Since recovery requires a team of multidisciplinary healthcare providers, family, and friends, each team member needs to see, understand, and treat the patient as an individual to motivate them to move.

##### 3.2.5.1 Individualized care

Recognizing the patient as a person and how mobility impacts their life was an important way to individualize care and support early mobility. “It's (movement) a priority for me. I live on acreage, I have to maintain, and if I can't maintain it then, you know, my whole lifestyle is going to change so…so I was fighting to maintain a lifestyle. That wasn't looking too good with the situation I was in…” (P2). Individualized care also included how post-CS care needed to be tailored to the type of surgery done. One PWLE, who had an alternative access approach to CS, described this: “Everybody assumed I had the, chest (open sternotomy), so everyone was like “cross your arms cross your arms,’ and I was like no, I don't have that” (P7).

##### 3.2.5.2 Support & kindness from healthcare providers and care partners

A simple gesture that recognizes the patient as a person was supportive in helping PWLE get moving after surgery. P10 included a cup of coffee on his body map. When asked about this he shared:

“That's Hector, the orderly, yeah. Horrible food and the nurses told me you know what, Hector can make you real coffee and Hector brought me coffee in the morning and it was so good, it was real good coffee. Anyways so that that's my ode to all of the caregivers”

Care partners, such as family, were also important sources of support and kindness. They could help remind patients of what was waiting for them at home. One PWLE described the impact of this support and kindness: “She (their care partner) was there being positive and she would just talk about home and the cat, so you know, the normal things that we were doing…so that was very helpful and in and making sure that I move forward to… that was a key part” (P9).

## 4 Discussion

Exploring the experience of early mobility in the CSICU through PWLE engagement in creating body maps yielded themes and categories that describe an embodied experience of surviving CS. The use of an arts-based research method for PER allowed PWLE to share their early mobility experiences in a personal way, conveying their senses, positive and negative emotions, and feelings to shift our previous understanding of mobility and its meaning and significance to PWLE having CS. An integrated analysis of visual and verbal data sources generated five themes and 12 categories. The meaning of movement that extends beyond the physical is the central theme. Embodied experiences highlighted that early mobility pushes patients towards mobilizing and pulls them towards being sedentary through the paradox of moving, being treated as a person first, moving through the emotions, and attitude. The embodied experience of early mobility reveals the complexities and tensions experienced in recovery from CS when considering the imperative attributes of mobility during the post-operative period. Having access to PWLE embodied knowledge of what mobility is like, what it feels like and the inherent meaning(s) that motivate one to engage in their surgical journey is essential in providing relational CS care. These findings more fulsomely describe the experience of early mobility in the CSICU, which may support clinicians and researchers in better supporting early mobility practices in the CSICU, integrating the patient experience.

### 4.1 Embodied experiences of early mobility

Exploring the embodied experience revealed several novel elements of early mobility in the CSICU. Using arts-based research methods for PER revealed the significant meaning of mobility for patients following CS, highlighting the importance of being seen as a person rather than just a patient. Also described were the positive emotions associated with early mobility in the CSICU and the need to feel mentally prepared for mobility following cardiac surgery. These elements are embodied knowledge, as they are emotions and feelings that reside in the body and thus cannot be expressed only in words. These are an outcome of engagement, as without facilitating PWLE to share their experiences, questions related to these would not have been considered by the research team. This allowed us to describe the meaning attributed to moving, as described in the overall theme, making meaning of movement.

Moving through the emotions related to early mobility has been described by one other study more narrowly (58). The emotions discussed in that study most closely reflected the categories of being restricted and redefining self, as the authors described a mix of emotions experienced while patients struggle to retain independence and normal life through early mobility (58). They specifically mention that patients felt unprepared for the effort, weakness, and perceived lack of control they experienced (58). These authors also shared how patients felt helpless as they required healthcare providers to support them in mobilizing (58). This view limits the emotions to seemingly negative ones, missing the category happy to be alive. This balance in emotions is an important contribution of the current work and is another engagement outcome. Understanding the positive emotions associated with early mobility helps researchers and healthcare providers better connect with patients and support them in their early mobility journey. The paradox of movement and the two categories related to it, the tensions around moving and symptoms, are described in the literature. The tensions related to ICU equipment have been noted as a barrier to mobility by others, with suggestions from clinicians to mitigate these challenges (59). These suggestions included securing equipment and lines, using portable monitors, taking a step-wise approach to mobility, and clearly planning the mobility steps prior to mobilizing (59). Pain is a commonly described symptom related to early mobility following CS (58–61); and was noted to occur with early mobility even in cardiac ICU patients not undergoing CS (61). This work aligns with our study, as surgical site pain is only one type of pain that may be experienced. Premedicating patients to prevent pain is one potential solution (59). Another is to discuss pain with patients and share a study that found that engaging in early mobility reduced pain scores (60). Dubb and colleagues were the only researchers to describe the symptoms described in the current study (58). Feeling weak, foggy or fuzzy, and hallucinating were symptoms described in another study (58). Dizziness was experienced by cardiac ICU patients not undergoing CS (61), suggesting that this symptom may relate to more than only the CS. This combination of physical, cognitive, and mental symptoms reinforces the complexity of early mobility in the CS ICU and the need to seek ways to support patients through the process holistically.

The theme of attitude and its category of positive attitude have been noted in the literature. Early mobility may also cause or improve a positive attitude. Participants in one study shared that they felt they were recovering and felt less ill when they began moving (58). Attitude has been noted outside of the CS ICU population. In a study of patients with hip fractures, being positive and having a “good attitude” supported recovery of mobility (62). The category if you know, you can endure is a novel finding of this study. Some studies do explore knowledge related to mobility (59–61). However, these studies miss the patient perspective of feeling mentally prepared to endure mobilization. This important perspective needs to be integrated into education to help prepare patients and care partners for early mobility.

The category if you know, you can endure is a novel finding of this study. Some studies do explore knowledge related to mobility (59–61), however, these studies miss the patient perspective of feeling mentally prepared to endure mobilizing. This important perspective needs to be integrated into pre-operative education to help prepare patients and care partners for early mobility. This education could focus more on developing coping strategies for the potential difficulties of early mobility, such as pain. Pain is a commonly described symptom related to early mobility following CS (58, 60, 61). Discussing pain with patients and sharing that engaging in early mobility can reduce pain scores (60) may support patients in mentally preparing for pain and how they can overcome it in the moments of early mobility.

The theme person first is a novel finding for early mobility in the CSICU but aligns with the movement toward patient-centred care in healthcare (63). When applying knowledge about early mobility for patients undergoing CS, clinicians should consider integrating a patient-first approach in consultation with the person undergoing surgery. In this way, incorporating patient-related mobility goals, grounded in their meanings and connections to their pre-operative lives, would be the starting point for executing the plan for mobility post-CS, linking mobility to relevant outcomes that will motivate the patient to achieve evidence-based and guideline-supported post-operative mobility goals. Messaging about the importance of early mobility following CS can be tailored to patients by linking to what is meaningful to them and by focusing on the person first.

### 4.2 Implications

This research has clinical, research, and theoretical implications. Understanding the embodied experience of the meaning behind movement is important for researchers and healthcare providers. This can be leveraged to support the successful development and implementation of future clinical interventions that consider PWLE contexts.

Clinically, the findings can guide patient care and support the success of early mobilization in the CSICU by expanding the lens through which we look at early mobility. We intentionally expand our lens through the use of aesthetic knowing (64). Early mobility is more than an essential, post-operative guideline-based care requirement. What our findings reveal is that mobility, as seen, felt, heard, and communicated in an artistic process, extends beyond the empirical words and text representations of mobility. Early mobility is grounded in relational care. Mobility exists in concept and in its operationalization that involves the intricate interrelationships across person, significant others, professionals, and in the healthcare context. Therefore, clinicians can use these findings to better understand and relate to patients, to motivate them, and form more meaningful therapeutic relationships. This may support the success of early mobility. For example, rather than viewing early mobility as purely physical, we now begin to understand the emotions and meaning that patients impart on mobility. It may be prudent to enter a reciprocal partnership with the patient when devising a post-operative mobility plan that has been tailored by, for and with the patient's goals in mind.

These findings also have research implications, as the data indicate that using arts-based research methods for PER can elicit embodied knowledge from PWLE. We assert that engaging in inquiry that purposely integrates an arts-based approach to clinical research acknowledges embodied knowledge as a viable counter narrative to complement contrasting quantitative empirical evidence (64). The emotion and meaning patients place on early mobility nudges researchers to consider outcomes beyond the physical when evaluating early mobility interventions. This shift to exploring broader outcomes for interventions refocuses researchers on the need to take a holistic approach to healthcare and interventions. Incorporating the lived experience in intervention development can help researchers understand the impacts of the interventions and how to evaluate them appropriately. This challenges the dominant biomedical paradigm through which health research is typically conducted. Researchers need to be open to all sources of knowledge, such as the lived experience, and incorporate these into their work. This will support research directed at priorities and outcomes that patients, who are the end users of the research, deem important (16).

Finally, this research has theoretical implications. These findings challenge the predominance of a biomedical lens in clinical health research (65–68). Research conducted from this worldview assumes that knowledge is contextual, shaped by inclusion and exclusion criteria (66, 69). This means that a single reality and objective truth are available through rigorous research methods (68, 69). A biomedical worldview does not value subjective (66, 67) or aesthetic knowledge (12). While research through this worldview has made valuable contributions to healthcare (18), the underlying assumption of a single reality and the limited knowledge sources considered limit the contributions to clinical interventions (66–68). An alternative guiding worldview is required to explore and include the lived experience of patients and their care partners as a source of knowledge and to use subjective and aesthetic knowledge to inform clinical research (47, 70, 71). Pragmatism is such an alternative worldview.

Through a pragmatic worldview, knowledge is contextual and comes from experience (46, 47). For research to truly be directed at improving patient outcomes, those with experience, patients and care partners, must be engaged in the research. Research through a pragmatic worldview aims to produce logical findings relevant to the population and rational for the research (44–46). This aligns with a goal of PER (16). Through a pragmatic worldview, the best available research methods are used to explore the problem (46, 47). This supports the use of arts-based research methods for PER. Arts-based research methods facilitate dialogue and help build relationships among team members (12). Dialogue and power are integral to PER (49). Using arts-based research methods allows the exploration of subjective and aesthetic knowledge, leading to research through a pragmatic worldview to become anti-oppressive and emancipatory (72). This alignment of PER and arts-based research methods with a pragmatic worldview may support the use of this worldview in future research to better direct clinical research at addressing relevant problems using relevant and important sources of knowledge.

### 4.3 Strengths and limitations

This work has several strengths. First, there is a strong framework underpinning the approach to engagement (1). This is a strength because it provides an evidence-informed foundation for the project. Second, a layered data analysis occurred using both visual and narrative methodologies. This layered approach helped to overcome the limitations of each independent method. Such an analysis yields rich, reliable, and robust results. Third, the research team includes two PWLE who collaborated from ideation to dissemination as equal team members.

This work has limitations. First, there was a relatively small number of PWLE engaged as consultants. Engaging a more diverse group of PWLE consultants would have likely introduced a broader range of potentially unique perspectives. Second, all PWLE underwent CS at the same hospital, which also limits the generalizability of the findings. Third, due to the small group size, diversity beyond self-identified gender, role (patients or care partners), and geographic location was not possible. We recognize the limitation of this, and future research will intentionally focus on recruiting based on ethnic diversity in meaningful and culturally sensitive ways. This approach will support the development of an understanding of the needs of patients and care partners of different ethnicities rather than comparing the perspectives of patients and care partners based on ethnicity. Finally, the low number of care partners engaged and their preference to co-create body maps with their loved ones limits the understanding of their experiences. These should be explored in future studies.

## 5 Conclusion

Body mapping revealed aspects of their embodied experiences of early mobility that could not be explored through words alone. Embodied experiences are best explored using arts-based research methods, as they involve senses, expressions, and emotions that come from and reside in the body and cannot be verbalized. This is important knowledge to consider, which is overlooked when PER focuses only on verbal communication. Expanding PER methods to include arts-based research methods can deepen understanding of the embodied experiences of PWLE, ultimately enabling improvements to the healthcare system and patient health outcomes.

## Data Availability

Data are available from the corresponding author upon reasonable request.

## Supplementary material

The Supplementary Material for this article can be found online at: https://www.frontiersin.org/articles/10.3389/fresc.2026. 1801092/full#supplementary-material
